# Juvenile detachment, an early sign of departure from parental care, in the leech *Orientobdelloides siamensis* (Oka, 1917)

**DOI:** 10.1371/journal.pone.0302921

**Published:** 2024-11-25

**Authors:** Poramad Trivalairat, Krittiya Trivalairat, Tashfia Raquib, Watchariya Purivirojkul

**Affiliations:** 1 Princess Agrarajakumari College of Nursing, Chulabhorn Royal Academy, Bangkok, Thailand; 2 Department of Zoology, Animal Systematics and Ecology Speciality Research Unit (ASESRU), Faculty of Science, Kasetsart University, Bangkok, Thailand; 3 Department of Zoology, Laboratory for Exploring the Ectoparasites and Carnivorous Hirudineans (L.E.E.C.H.), Faculty of Science, Kasetsart University, Bangkok, Thailand; 4 Lee Kong Chian Natural History Museum, National University of Singapore, Singapore, Singapore; Universidade Federal de Minas Gerais, BRAZIL

## Abstract

Glossiphoniidae is a family of freshwater leeches, notable for their unique behaviour of parental care. After hatching, juveniles remain on the ventral side of their parent, where they receive protection and grow until they are ready to depart from the parent leech. The detachment of juveniles is a crucial stage for their development and independence from their parents, potentially influenced by various factors. To investigate these factors, ten parental individuals of *Orientobdelloides siamensis* were studied in the laboratory. Three to five days after copulation, all parental leeches deposited approximately 361.6±37.79 eggs on the substrate, which were covered until the end of the brooding period. Incubation of their single-egg cocoons took 7–9 days. Subsequently, the newborns attached to the ventral annulus of the parent by their caudal sucker. Seven to eleven days after hatching, the caudal sucker of juveniles expanded over the parent’s annulus, indicating readiness to depart. The young leeches detached from the parental venter, moved to the substrate, and continued living under the ventral side of their parent. Finally, to determine the timing of juvenile detachment, the space availability beneath the parental venter and yolk depletion after hatching were analyzed. By observing morphological characteristics and behaviors, this study was able to investigate the interaction between these factors, and their correlation with juvenile detachment in *O*. *siamensis*.

## Introduction

Glossiphoniidae Vaillant, 1890, is a family of freshwater, proboscis-bearing leeches distributed worldwide [1, 2]. Besides being distinguished by morphological characteristics, Glossiphoniidae also exhibit a unique parental care behaviour, in protecting and feeding of their young beneath the ventral surface [1–5]. They are categorized into three subfamilies on the basis of cocoon attachments: Glossiphoniinae (e.g., *Glossiphonia complanata* Linnaeus, 1758), which attach cocoons to substrates, Haementeriinae (e.g., *Helobdella stagnalis* Linnaeus, 1758; *H*. *triserialis* Blanchard, 1849), which attach cocoons directly to the parent’s ventral surface, and the monogeneric Theromyzinae (e.g., *Theromyzon tessulatum* Müller, 1774), which exhibit a combination of these traits [4–10]. The placement of the cocoon on the substrate represents a primitive characteristic of this annelid worm, reminiscent of ancestors in polychaetes, oligochaetes, and even primitive leeches (Infraclass Acanthobdellida) [2, 4]. Furthermore, each species of glossiphoniid leech has a distinct brooding period that ranges from 22 to 165 days, beginning from egg deposition to maturity [11, 12]. However, regardless of the species-specific lengths of brooding periods, juveniles of these species depart from their parents punctually within a single day. While some young leeches may experience yolk depletion and briefly separate from their parents, as observed in *G*. *complanata*, others, such as *H*. *stagnalis*, have a longer brooding period, by nourishing their young with a dietary meal until they fully mature [4]. Aside from yolk storage, what are the other factors that may influence the timing of departure from the parental leech?

Currently, there are four reported glossiphoniid leech species in Thailand: *Orientobdelloides siamensis* (Oka, 1917) (formerly *Placobdelloides siamensis*); *O*. *sirikanchanae* (Trivalairat et al., 2019) (formerly *P*. *sirikanchanae*); *O*. *tridens* (Chiangkul et al., 2020a) (formerly *P*. *tridens*); and *Batracobdelloides bangkhenensis* Chiangkul et al., 2020b [12–15]. Most of these species are ectoparasites feeding on geoemydid terrapins, except for *B*. *bangkhenensis*, which preys on freshwater snails [2, 5, 12–15]. *Orientobdelloides siamensis*, a member of the Glossiphoniinae subfamily, is the only species for which a complete life cycle and conditions have been reported. It has the shortest life cycle, lasting no more than a month from hatching to maturity, as well as the highest number of eggs per clutch and a large enough body size for observation with the naked eye [12]. *Orientobdelloides siamensis* serves as a suitable model for examining parental departure to enhance our understanding of the departure of young leeches unaffected by parental nourishment. Therefore, we conducted an observational study to compare the caudal sucker diameter of juveniles and parent ventral annuli, the body size difference between juveniles and parents, and the remaining yolk in the crop caeca in relation to departure behaviours.

## Materials and methods

### Specimen preparation

Ten six-month-old (mature) specimens of *O*. *siamensis* were obtained from the laboratory of the Department of Zoology, Faculty of Science, Kasetsart University, Bangkok Province, Thailand (13° 50’ 53.6" N 100° 33’ 47.3" E), which were descendants from a previous study by Chiangkul et al. (2020). They were introduced into a large container measuring 57x32x33 cm³, that housed a Malayan snail-eating turtle (*Malayemys macrocephala* (Gray, 1859)), with water filled to two-thirds of the turtle’s height, to encourage feeding and copulation [16]. After copulation, each specimen was isolated without food in a small container measuring 12x12x15 cm^3^ to facilitate egg deposition and raising of juveniles (S1 Video). The water temperature in all containers was recorded every six hours at 0000, 0600, 1200, and 1800 hours daily. Throughout the observation period, 1 February to 21 March 2024, the mean water temperature in the leech container was 29.1±2.4°C.

**Fig 1:**
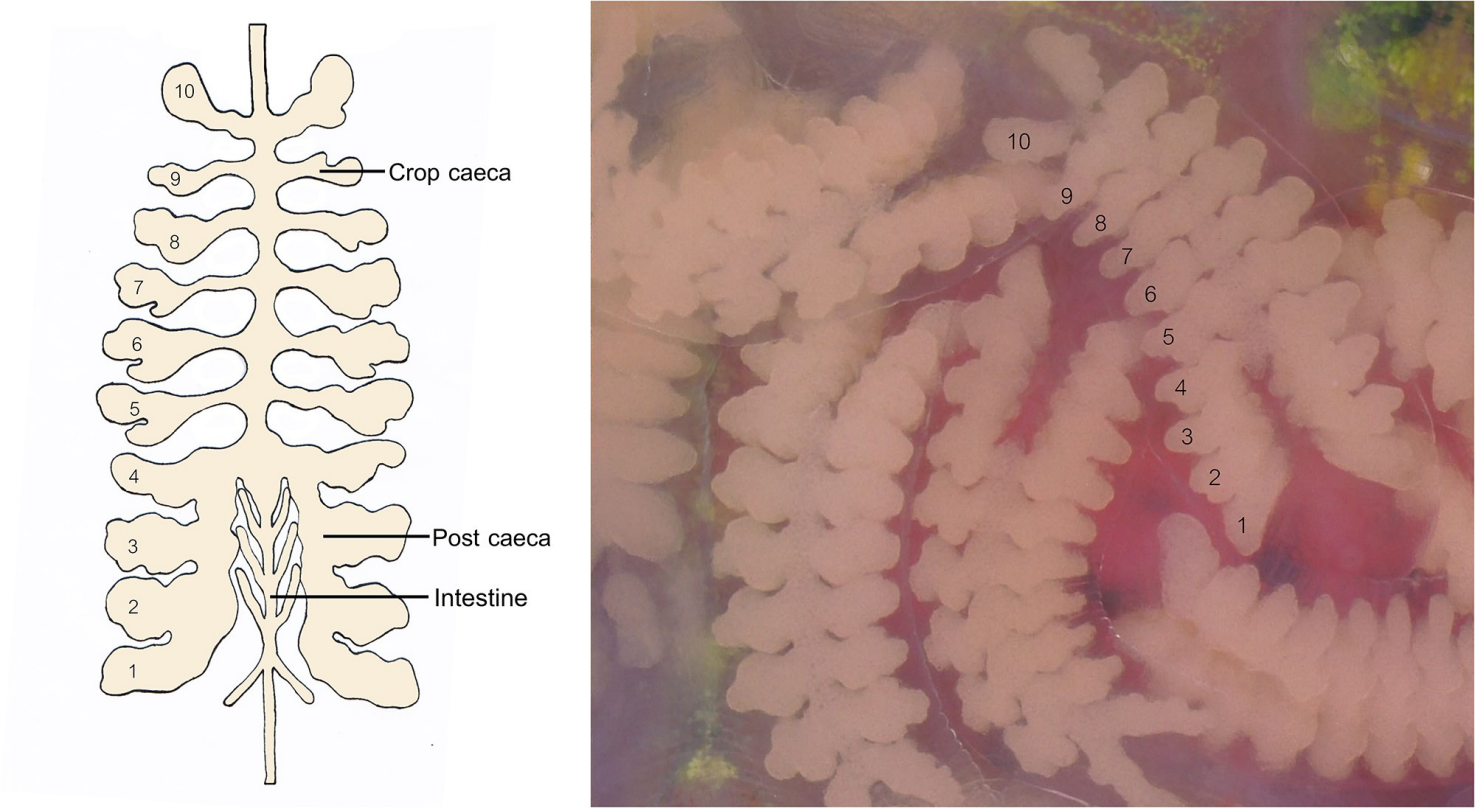
Yolk score assessment on a scale of 1 to 10, ranging from the first anterior crop caeca to the fourth branch of the post-caeca.

Upon hatching, the number of juveniles from each parent was counted and their crop caeca were observed daily for yolk storage until they left the parent. Yolk storage was assessed on a scale of 0 to 10, where 10 indicated full egg yolk in all crop caeca (comprising 6 anterior crop caeca and 4 post-caeca branches) and 0 indicated complete depletion of yolk in all crop caeca (Fig 1). The score was reduced by one when yolk in the crop caeca was depleted from anterior to posterior.

Additionally, ten cocoons or juveniles from each parent were collected daily as well as all parents from the last day that the juveniles completely departed. These specimens were fixed in 4% glutaraldehyde for subsequent examination by scanning electron microscopy (SEM) and in 10% neutral buffered formalin for histological analysis.

### Preparation for scanning electron microscopy

The specimens preserved in 4% glutaraldehyde were dehydrated in a series of ethanol solutions (70%, 80%, 95%, absolute ethanol) and spent two 1-hour cycles in each concentration. This dehydration procedure was conducted using a critical point dryer (CPD; model HCP-2) until the specimens reached critical dry point. The specimens were placed onto a stub using carbon tape and then sputter-coated with gold particles. All coated specimens were examined using a scanning electron microscope (SEM) to count the parent ventral annuli; measure body length and width, including the length and width of the cocoon; and determine the diameter of the oral and caudal sucker of both parent and juvenile leeches.

Additionally, the capacity of offspring beneath the parent venter was calculated by multiplying three-fourths of the parent’s body area by the offspring’s body area each day. As *O*. *siamensis* was determined to have an oval shape, body area was calculated using the oval formula (π x (body width/2) x (body length/2)) for both parents and offspring.

### Preparation for histological studies

**Fig 2:**
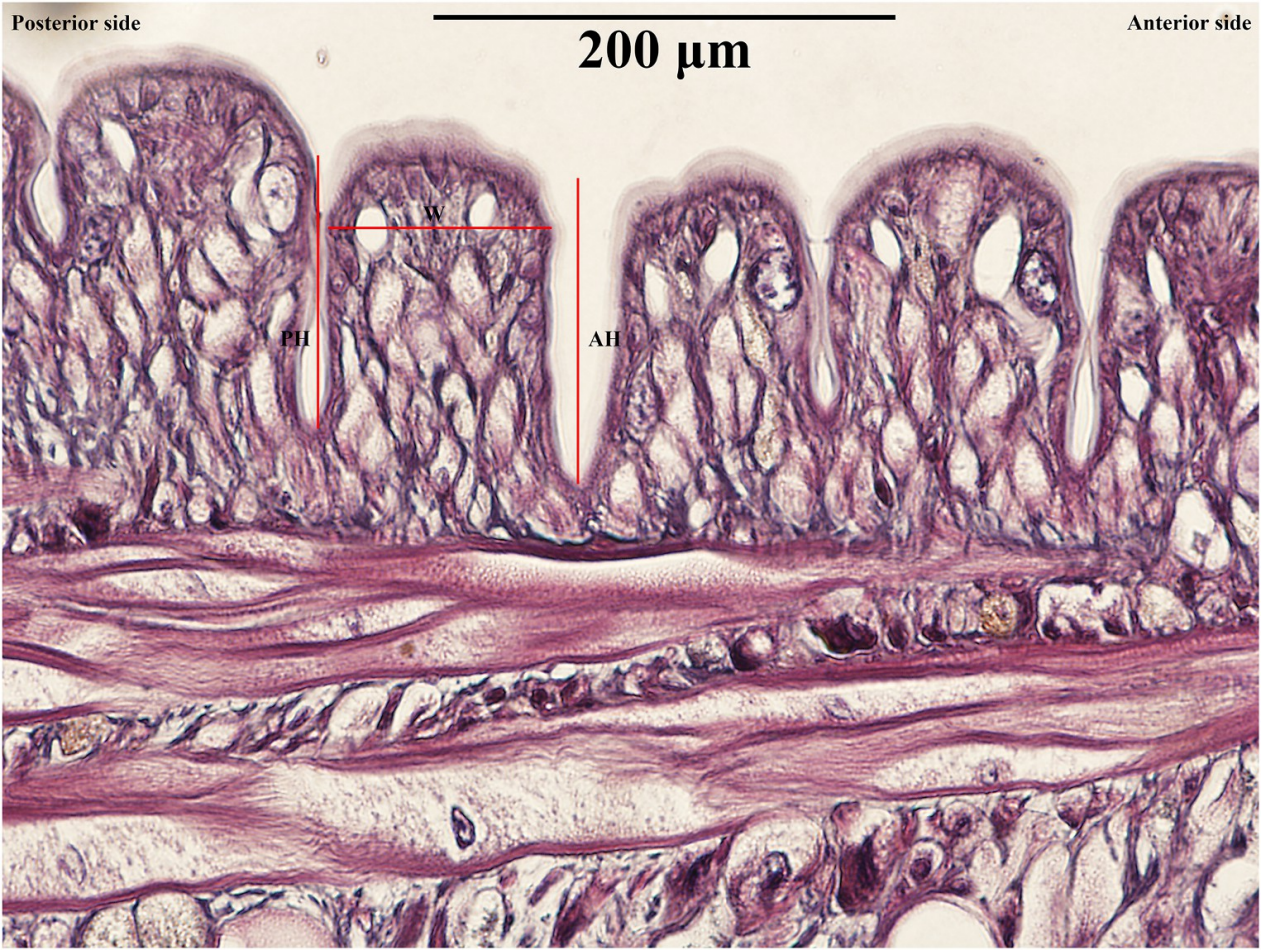
Measurements of annuli in *Orientobdelloides siamensis*: Anterior height (AH); posterior height (PH); and width (W).

The specimens were preserved in 10% NBF for 12 hours before dehydrating through a series of ethanol solutions (70%, 80%, 90%, 95%, and absolute ethanol). They were cleared in xylene and embedded in paraffin blocks. The specimens were then sectioned into 5 mm-thick serial longitudinal sections using a rotary microtome. Following sectioning, the specimens were stained with hematoxylin and eosin (H&E) and mounted with Permount. The histological slides were examined under a light microscope to measure anterior height (AH), posterior height (PH), and width (AW) to sum up the cross-sectional area (CSA) of each annulus (total 76 annuli) (Fig 2). All photomicrographs were processed using Photoshop CS6.

### Ethics statement

This research was approved by the Institute of Animal Care and Use Committee at Kasetsart University under number ACKU66-SCI-016.

## Results

### Pre-hatching

**Fig 3:**
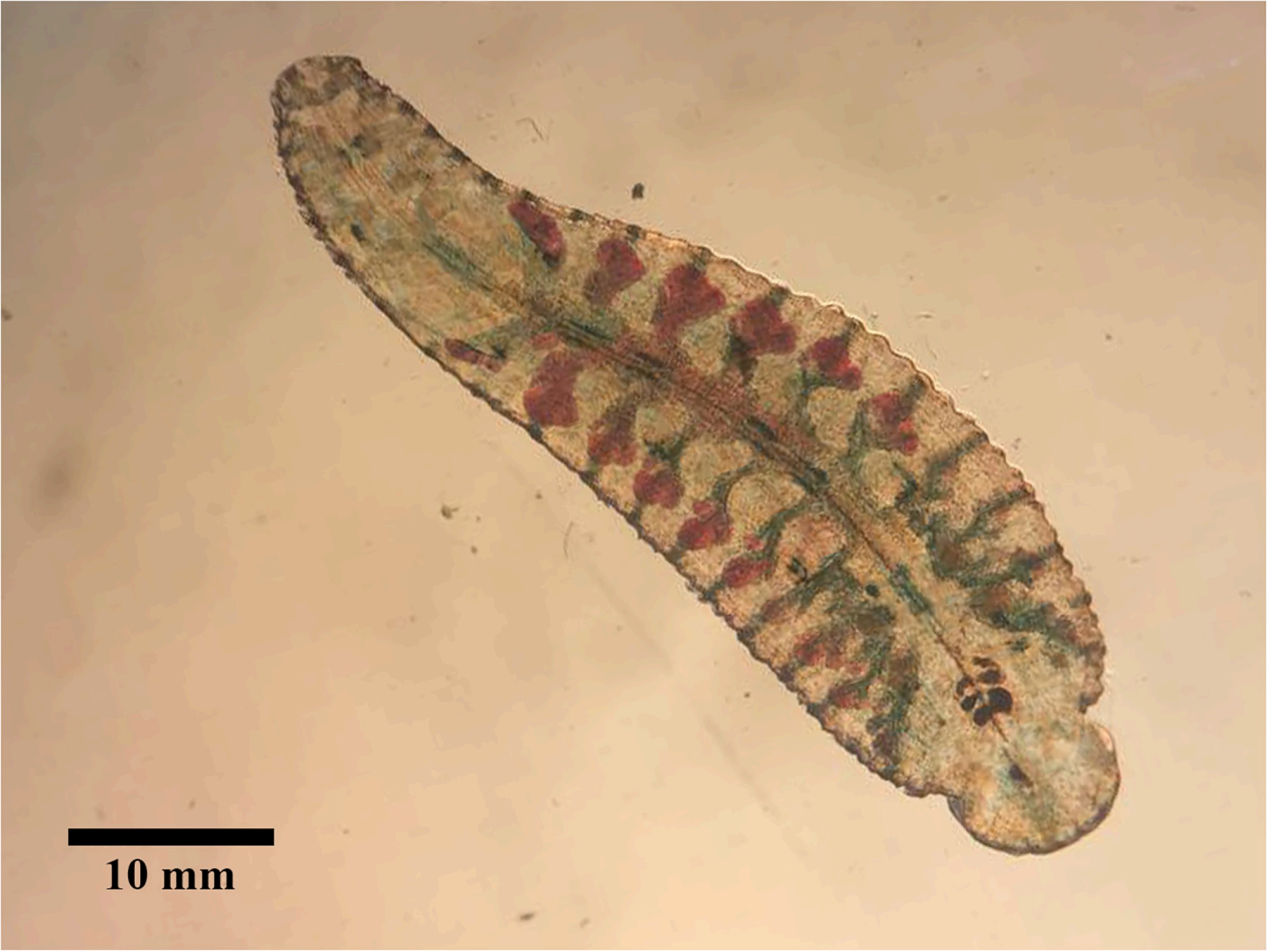
Mature *Orientobdelloides siamensis* exhibiting blood within the crop caeca.

**Fig 4:**
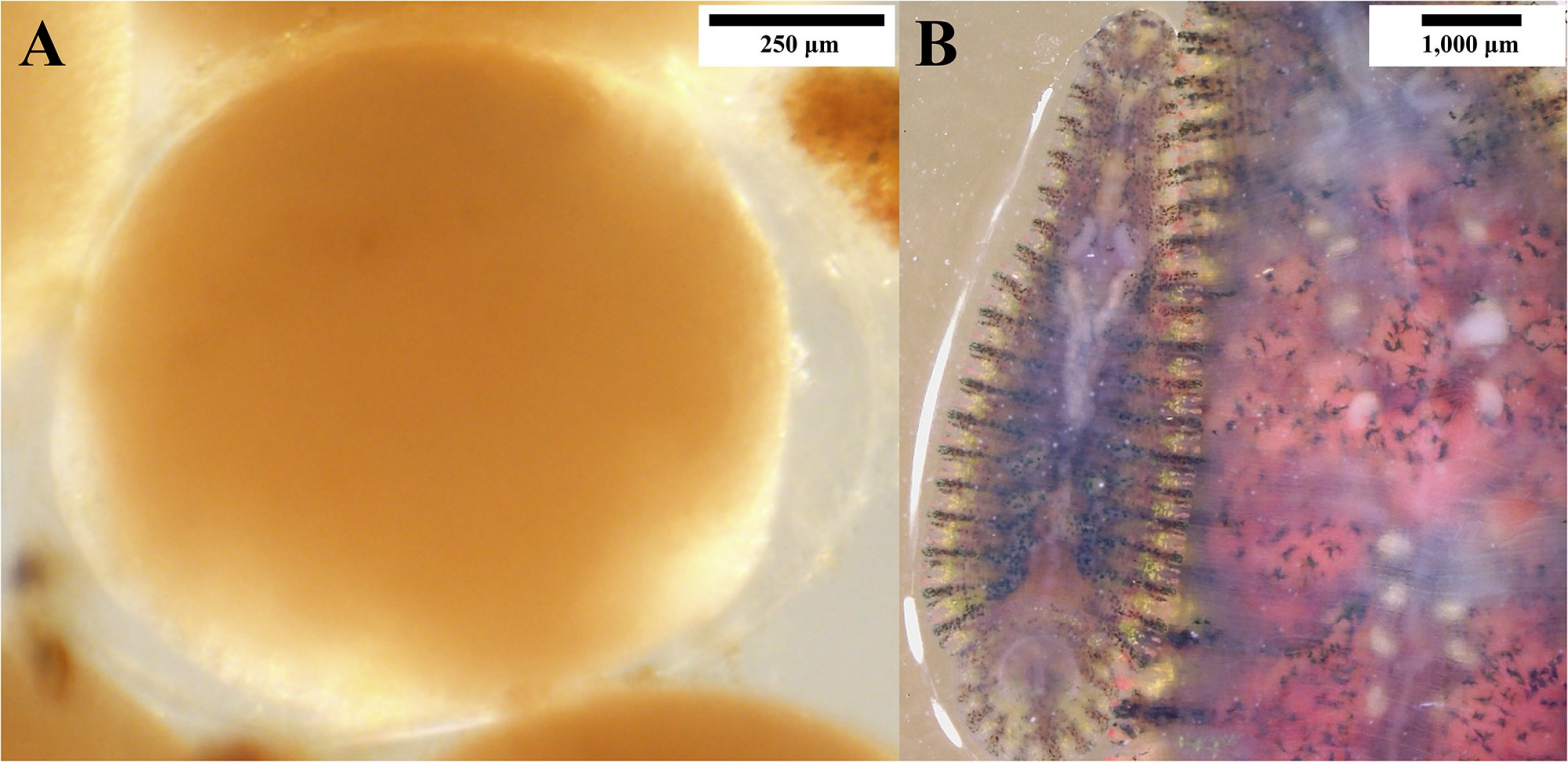
(A) Translucent cocoon containing an egg and (B) twenty-one-day-old juvenile of *Orientobdelloides siamensis*.

Before the experiment, all ten six-month-old *O*. *siamensis* individuals were starved for two weeks. On 17 February 2024, they were introduced into a large water container with a Malayan snail-eating turtle to feed and stimulate copulation (Fig 3). These leeches are active during the night. After two days, on 19 February 2024, all leeches had white creamy eggs inside their oviducts, and crop caeca containing turtle blood. At this point, they were transferred to separate small containers for isolation. Most individuals deposited a clutch of cocoons between the third and fifth days following isolation (22 to 25 February 2024). These were single-egg cocoons, with an average clutch size of 361.6±37.79 eggs (range: 317–415 eggs, n = 10), average cocoon length of 455.75±36.53 μm (range: 363.28–564.97 μm, n = 100) and cocoon width of 409.33±39.12 μm (range: 298.46–478.14 μm, n = 100) (Fig 4) (S1 Table). Ten cocoons were collected from each parent for analysis. The eggs turned a creamy brownish color during the fifth and sixth days after deposition (27 February to 2 March 2024) and hatched within a few days thereafter (29 February to 5 March 2024).

### Post-hatching

**Fig 5:**
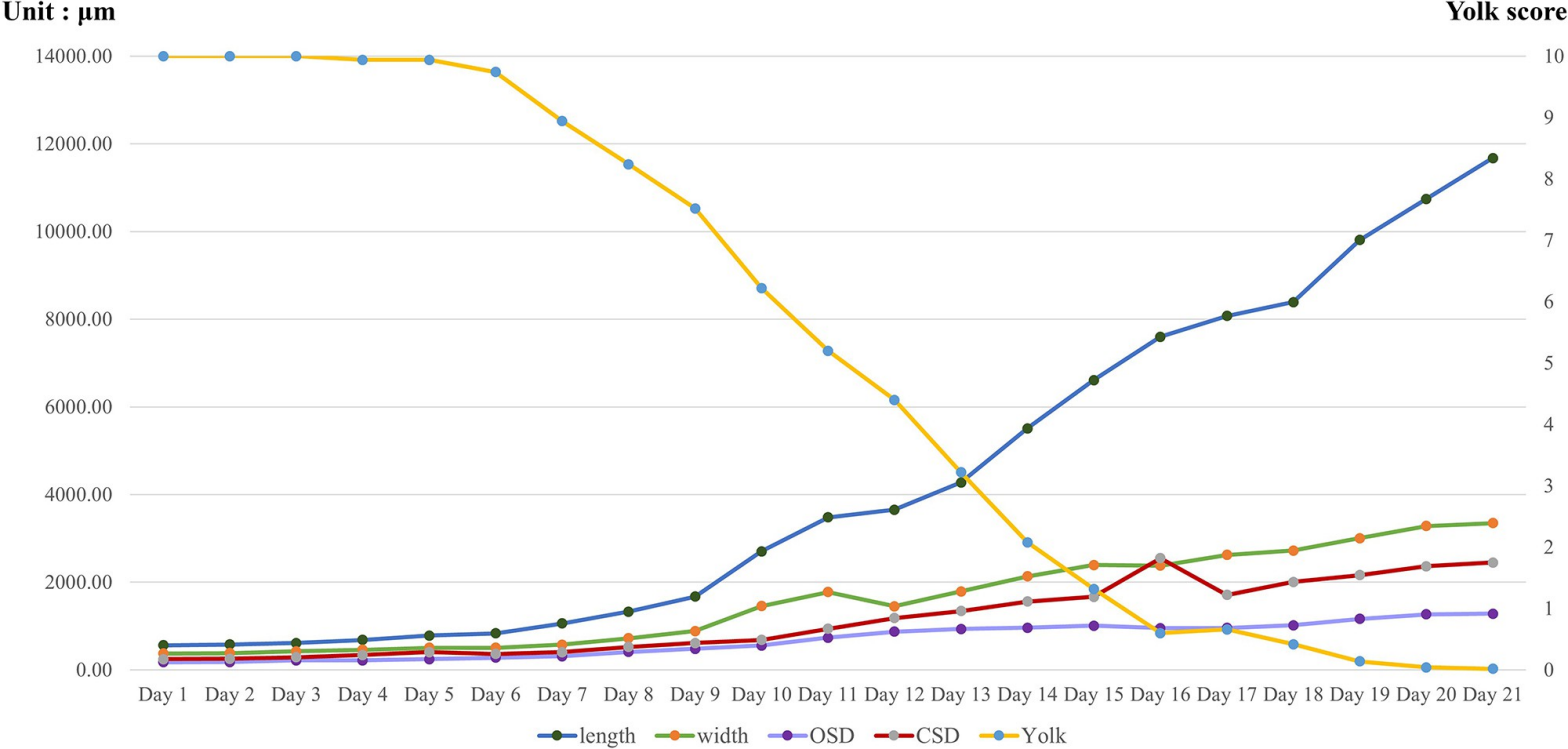
Growth rates of various characteristics, including body length and width, oral sucker diameter (OSD), and caudal sucker diameter (CSD), in juveniles of *Orientobdelloides siamensis*, alongside the rate of yolk depletion.

Newly hatched juveniles attached to their parent’s ventral surface for the first week after hatching. They began to detach from the parent between days 7–11 and move to the substrate underneath the parent’s body where they stayed until the end of the second week, before departing from the parental leech. Throughout this transitional phase, the juveniles demonstrated a significant increase in body dimensions with lengths ranging approximately 1,059.60–3,480.97 μm (1.88–6.18 times greater than hatching day), widths ranging 576.47–1,778.29 μm (1.54–4.74 times), oral sucker diameter (OSD) varying between 314.59–737.95 μm (1.79–4.20 times), and caudal sucker diameter (CSD) ranging 408.86–936.71 μm (1.66–3.79 times) (Fig 5) (S1 Table).

In comparison, the adult leeches had an average body length of 53,802.04±5,632.71 μm (range: 49,232.57–61,045.56 μm), a width of 26,475.53±4,609.98 μm (range: 21,218.91–32,175.33 μm), OSD measuring 9,147.65±2,715.93 μm (range: 6,169.15–12,542.45 μm), and CSD of 10,874.05±2,222.81 μm (range: 8,905.47–13,418.16 μm). A total of 76 annuli were categorized into anterior and posterior sections, with the highest attached annulus, the 30^th^ annulus of the parental leech, serving as the dividing point.

After the young leeches departed from their parents on the 21^st^ day post-hatching (21 March 2024), the parental leeches underwent fixation and histological processing to measure the total cross-sectional area (CSA) of each annulus by combining the widths and heights (anterior (AH) and posterior heights (PH)) of the annuli (as illustrated in S2 Table). Most anterior annuli exceeded a height of 100 μm, except those around the gonopore (99.35–144.30 μm). The male gonopore was commonly found between annuli 20 and 21, varying between 20/21, 22/23, 25/26, and 26/27, while the female gonopore was usually positioned between annuli 22 and 23, varying between 22/23, 24/25, 27/28, and 28/29. The posterior section, where the offspring were distributed, exhibited an average CSA of 432.90±68.31 μm (range: 280.36–611.98 μm), width of 261.77±67.08 μm (range: 127.95–383.86 μm), AH 81.25±27.63 μm (range: 29.22–130.46 μm), and PH 89.87±30.56 μm (range: 32.32–144.30 μm).

**Fig 6:**
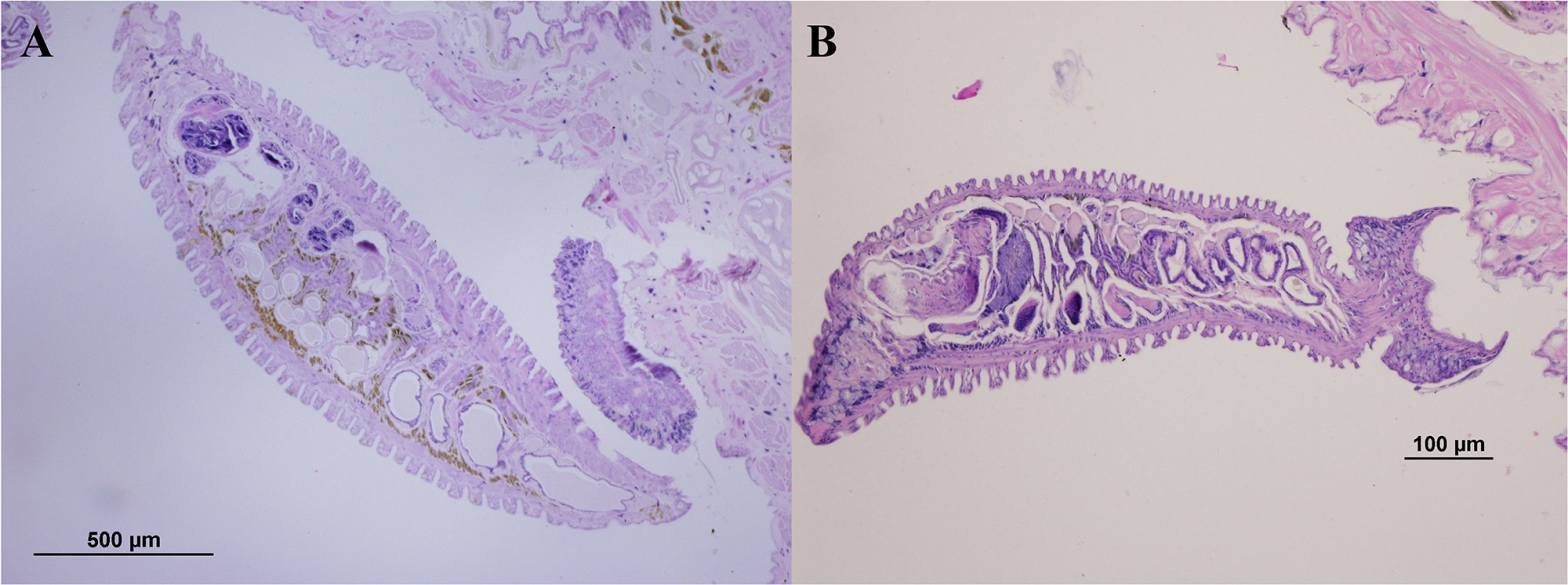
Attachment of *Orientobdelloides siamensis* juvenile by gripping on parent annuli: (A) five-day-old leech gripping onto the tip of the parent annulus and (B) ten-day-old leech gripping onto the whole parent annulus.

**Fig 7:**
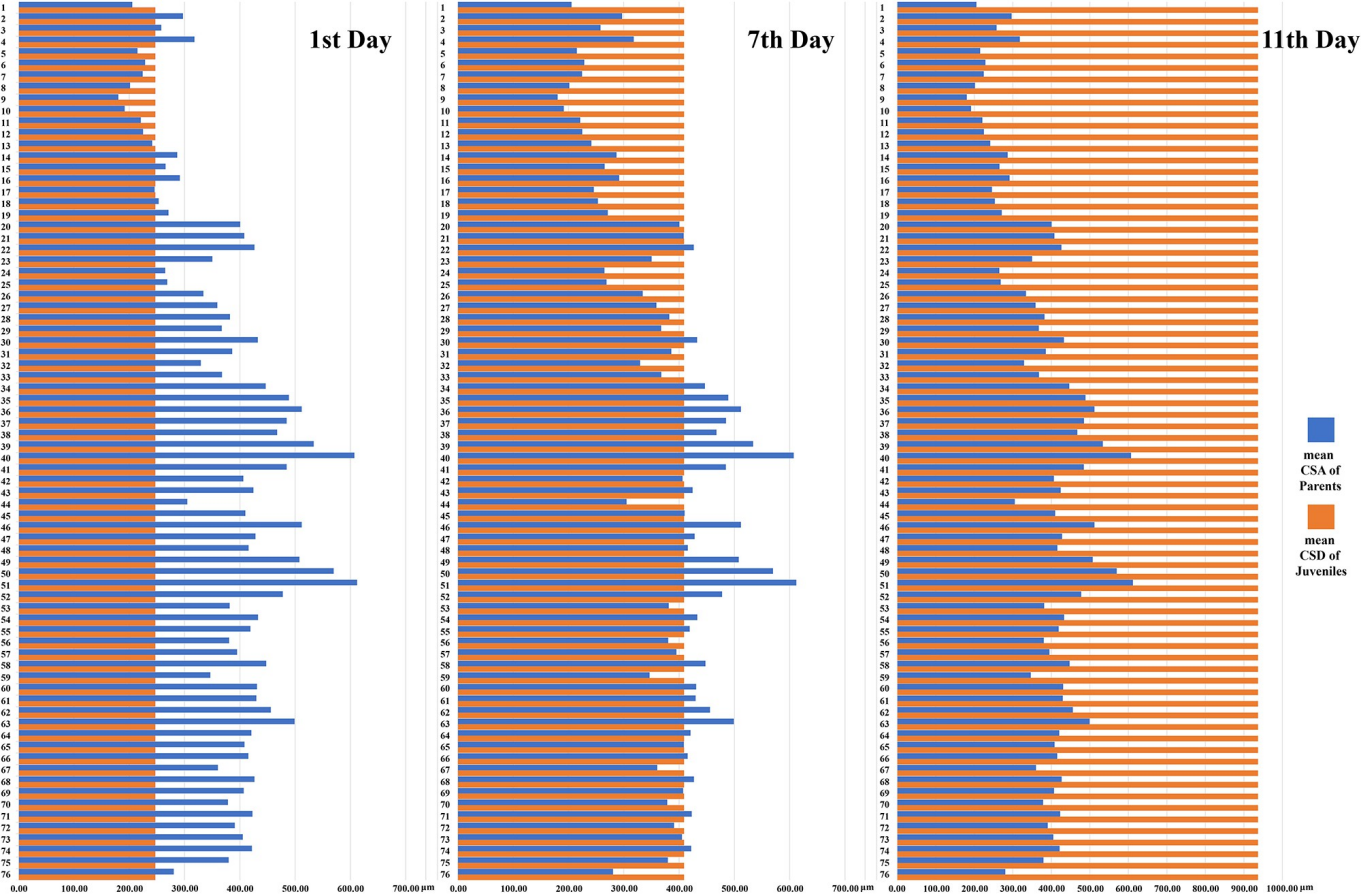
Comparison of parent annuli (annuli 1–76) and mean caudal sucker diameter (CSD) of juveniles in *Orientobdelloides siamensis* on the first, seventh, and eleventh days after hatching.

Seven days after hatching (7 March 2024), the juveniles began to shift their attachment from the ventral surface to the substrate. This occurred notably quicker when their average CSD surpassed their posterior CSA on the eighth day (526.16±81.13 vs 432.90±68.31 μm), although the precise number of attachment transitions could not be determined (Fig 6). All juveniles completely transitioned to substrate attachment by the eleventh day (11 March 2024), with CSD nearly doubled compared to three days earlier (936.71±162.51 μm, ratio 1.72) and 3.66 times greater than at birth (Fig 7).

Additionally, on 10 March 2024, the average remaining yolk score decreased by almost half to 5.2. Four days later (14 March 2024), the first juveniles began to move away from their parent’s ventral surface as their yolk reserves depleted. These 14-day-old juveniles measured 5,510.72±749.83 μm (range: 4,164.59–8,129.68 μm) in length, 2,133.33±291.91 μm (range: 1,542.29–2,935.32 μm) in width, 964.41±139.52 μm (range: 711.74–1,281.14 μm) in OSD, and 1,558.01±224.43 μm (range: 1,103.20–2,206.41 μm) in CSD. Subsequently, other young leeches whose yolk reserves were depleted also commenced departure from their parents. The last day of co-habitation with their parents was when they reached twenty-one days of age (21 March 2024). On this day, their average body size was 11,676.25±1,764.47 μm in length (range: 7,603.06–16,988.09 μm), 3,351.13±691.35 μm in width (range: 1,841.69–5,059.99 μm), 1,283.55±375.59 μm in OSD (range: 607.70–2,651.80 μm), and 2,447.81±471.75 μm in CSD (range: 1,565.96–4,037.54 μm).

## Discussion

### Oviposition and taxonomic characterization

The behaviour observed in the glossiphoniid leech, *O*. *siamensis*, represents a transitional stage between the absence of parental care in erpobdellid or hirudinid leeches (Arhynchobdellida) and the developed parental care observed in Theromyzinae leeches, which possess a brood pouch for carrying and protecting cocoons and juveniles [4, 17–20]. This behavior of parental care in glossiphoniid leeches is most advanced in the subfamily Haementeriinae, which exhibits developed feeding behaviour towards their juveniles until they reach maturity.

A previous study by Chiangkul et al. (2020a) found that *O*. *siamensis* (formerly *P*. *siamensis*) deposited cocoons and brooded juveniles on the ventral surface of its body, like the behaviour described in subfamily Haementeriinae which typically broods the eggs/cocoons and juveniles by attaching them to the ventral surface [12]. This observation differed from the description in Sawyer (1986), which classified this species under the subfamily Glossiphoniinae on the basis of its cocoon deposition on substrates [2]. Our current study found that *O*. *siamensis* deposited tiny single-egg cocoons on the substrate (size of the cocoon compared to their parent is 455.75 vs. 53,802.04 μm in length, 409.33 vs. 26,475.53 μm in width) before the juveniles hatch and attach to their parent’s posterior ventral surface (around three-fourths of the body).

This observed pattern of brooding indicates that *O*. *siamensis* shares the strategies of parental care with members of Glossiphoniinae and Haementeriinae. This species, now classified under the subfamily Glossiphoniinae, deposits its eggs on substrates and aligns with the classification by Sawyer (1986), which encompasses a total of seven genera, including *Baicaloclepsis* Lukin and Epshtein, 1959, *Glossiphonia* Johnson, 1816, *Hemiclepsis* Vejdovsky, 1884, *Parabdella* Autrum, 1936, *Placobdella* Blanchard, 1893, *Torix* Blanchard, 1893, and *Orientobdelloides* [2, 21–27].

### Factors affecting juvenile detachment from parent

Typically, the caudal sucker of leeches plays a key role in adhering to the substrate through wet adhesive force (for smooth substrate) or contraction of muscle fibers (for rough substrate) [28]. Leeches use these adhesive properties in both their caudal and oral suckers, facilitating a distinctive mode of locomotion among Hirudinea. In this study, the newly hatched *O*. *siamensis* transitioned their attachment from the substrate to their parent’s ventral surface by using their caudal sucker to adhere onto the annulus. Initially, the diameters of most caudal suckers (CSD) in newborns were smaller than the cross-sectional area (CSA) in the posterior ventral region of the parent (246.87 vs. 432.90 μm). This disparity in size between the CSD and CSA facilitated their adherence onto the annulus. Despite the rough surface of the parent’s annulus, the CSD’s grip on the annulus tip expands and changes to grip the whole annulus (as illustrated in Fig 5). The caudal sucker consists of soft collagen tissues and highly ductile epidermal tissues which aid in adhesion [28, 29]. However, as the juveniles grew larger, their caudal sucker also increased in size until it surpassed the dimensions of the parent’s annulus. On the seventh day after hatching, some CSD measurements of the juveniles exceeded those of the parent’s annulus (408.86±52.27 μm, range: 305.41–551.35 μm), which corresponded with the juveniles transitioning their attachment back to the substrate. The last observed day of attachment to their parent was the eleventh day after hatching, with the caudal sucker almost quadrupling (3.66 times) in size (936.71±162.51 μm, range: 696.20–1,376.58 μm). In theory, larger caudal suckers may provide leverage and a greater range of motion in comparison to smaller caudal suckers, potentially reducing the amount of force required for gripping [28, 30, 31]. This would imply the caudal sucker is important for leech survival as its growth signals changes in juveniles and may facilitate their first departure from the parent by reducing the adhesive force needed for attachment.

Moreover, upon hatching, the newly hatched juveniles of *O*. *siamensis* exhibited a substantial size difference in comparison to their parents. Juvenile body length was nearly one hundred times smaller (95.51 times, 563.30 vs. 53,802.04 μm), likewise, body width, OSD, and CSD were smaller by 70.50 times (375.53 vs. 26,475.53 μm), 52.04 times (175.78 vs. 9,147.65 μm), and 44.05 times (246.87 vs. 10,874.05 μm), respectively. Large juveniles acquire high amounts of energy from their parents [32–34]. Some animals, including *O*. *siamensis*, might adapt to reduce this energy investment in offspring by decreasing the offspring’s size and increasing their number, thereby increasing their survival rate. However, these juveniles exhibited a rapid growth rate, with a 9.78-fold increase in length and 5.68-fold increase in width when they departed on the 14^th^ day (14 March 2024), as compared to the hatching day. They reached a remarkable 20.73-fold increase in length and 8.92-fold increase in width on the last day of departure (21 March 2024).

Examination of the parents’ oval-shaped body revealed the dimensions of the area beneath the parental venter which could accommodate all juveniles for a duration, based on a juvenile individual area of 166,205.72 μm^2^ on the hatching day. Remarkably, only three-fourths of the parent’s total body area (839,400,570.42 μm^2^) was utilized from the foremost attachment point (annulus 30), offering significant capacity for newly hatched juveniles, with a maximum of 5,050.37 individuals (the maximum number of eggs per clutch in this study was 415). As they grow, juveniles can adjust by overlapping with each other to conserve space, while their parents could expand their bodies to provide additional space. The results indicated that the space under the parental venter exceeded the capacity to accommodate all juveniles by the 10th day post-hatching (271.21 individuals), further decreasing to 27.30 individuals by the 21st day (the last day of observed parental care). However, observations revealed that juveniles had left the parent by the 14th day and had completely dispersed by the 21st day. Like other brooding animals, such as mouth-brooding fish where body size plays a central role in the juvenile’s decision to depart from the mother’s mouth, these observations highlight the relationship between the space capacity in the natal habitat (under the ventral surface) and the size of the juveniles at the point of departure from the parent [35].

Lastly, the storage yolk, a significant determinant influencing the departure of offspring, plays a crucial role in leech development. Leech yolk, which is rich in essential nutrients such as vitellin and vitellogenin proteins, serves as a primary source of sustenance for juvenile leeches prior to independent feeding [36, 37]. Among glossiphoniid leeches, *O*. *siamensis* stands out for its substantial investment in oogenesis, which is evident in the copious yolk reserves it allocates to its eggs. This species exhibits prolific reproduction, with clutches containing an average of 361.6±37.79 eggs (range: 317–415 eggs) observed in this study, demonstrating two to eight times higher fecundity compared to other species of *Orientobdelloides* and *Helobdella*, which typically lay clutches of 50–200 eggs [4, 14, 17–19, 38, 39].

In this study, storage yolk deposition was visualized through transparent crop caeca, a feature exclusive to juvenile leeches. Initially, yolk presence was scored at 10, denoting complete occupancy across all branches (six in the anterior caeca and four post-caeca). Over the course of ten days, yolk depletion paralleled body growth (as illustrated in Fig 3), where juveniles depleted approximately half of their yolk reserves (score 5.2), nearing complete exhaustion by the 21st day and coinciding with their departure.

*Orientobdelloides siamensis* notably diverges from typical reproductive strategies by extending parental investment beyond oogenesis [40]. While it lacks feeding behavior akin to that of *Helobdella* species, which hunt prey and feed it to their offspring [18], *O*. *siamensis* exhibits a unique reproductive strategy, parasitizing turtle shells for egg deposition, thus providing offspring with easier access to a blood meal without the need to search for a host later [4, 18, 41–43]. Hence, yolk provision represents a crucial parental investment in the initial growth and development of offspring, ensuring their survival prior to independent feeding. Moreover, the selection of suitable egg deposition sites enhances offspring survival by facilitating continued access to nourishment post-yolk depletion.

**Fig 8:**
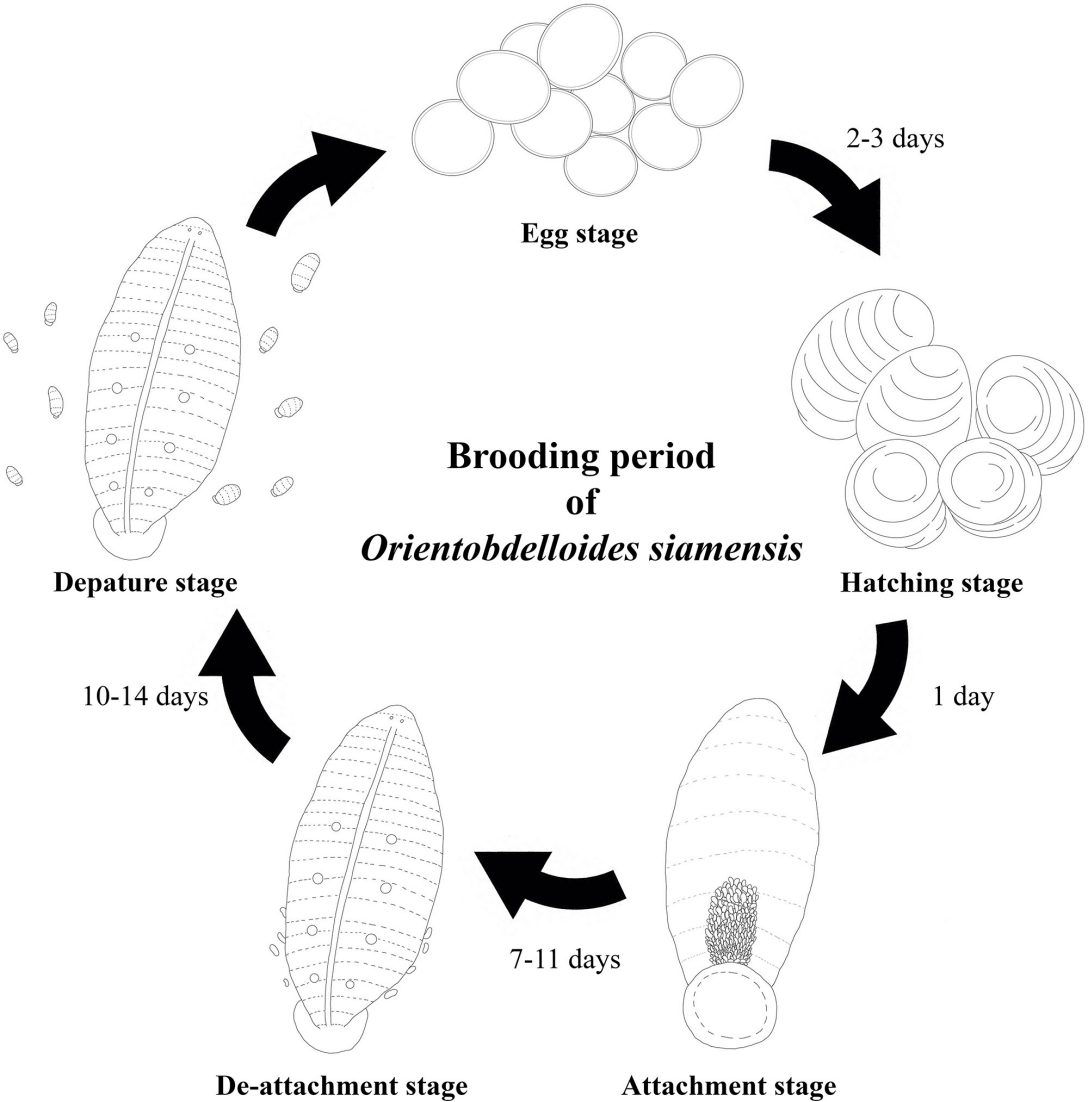
Brooding period of *Orientobdelloides siamensis*.

In summary, the departure of juvenile *O*. *siamensis* during the brooding period appears to be governed by multiple factors. After hatching, the newborn juveniles undergo an attachment stage beneath the parent vent, which occurs around days 7–11 (Fig 8). This stage is characterized by their adherence to the parent’s ventral surface, facilitated by the caudal sucker. Following the attachment stage, the detachment stage begins between days 7–11 and lasts approximately till days 10–14 before the departure stage begins. During this phase, the expansion of the caudal sucker may enable detachment, signaling the juvenile’s readiness to depart. Subsequently, the departure stage ensues, which is marked by yolk depletion and the accommodation of juveniles as they mature. Additionally, parental care ensures survival, as shown by the deposition of eggs on turtle shells which provided access to nourishment. Collectively, these elements including caudal sucker development, yolk provision, and available space, may play pivotal roles in shaping the departure and survival of offspring.

## Declaration of generative AI and AI-assisted technologies in the writing process

During the preparation of this work the authors used ChatGPT to check grammar and improve language. After using this tool/service, the authors reviewed and edited the content as needed and take full responsibility for the content of the publication.

## Supporting information

S1 Table Measurements of various characteristics in *Orientobdelloides siamensis* juveniles from hatching to parental departure (Day 1–21). (DOCX)

S2 Table Characteristics and cross-sectional area of each annulus (1–76) from *Orientobdelloides siamensis*. (DOCX)

S1 Video Facilitate the raising of *Orientobdelloides siamensis* juveniles. (MOV)
