# Supplementary material for: Juvenile detachment, an early sign of departure from parental care, in the leech Orientobdelloides siamensis (Oka, 1917)
Source: PLoS One. 2024 Nov 25;19(11):e0302921. doi: 10.1371/journal.pone.0302921 (PMC11588279; doi:10.1371/journal.pone.0302921)
Supplement: S1 Table — (DOCX) [file pone.0302921.s001.docx]

**Supplementary Table 1** Measurements of various characteristics in *Orientobdelloides siamensis* juveniles from hatching to parental departure (Day 1-21)

| **Date** | **Length (L) (μm)**  **(min-max)** | **Width (W) (μm)**  **(min-max)** | **L/W** | **Oval area (sqμm)**  **(3/4 Parent area/ Juvenile area)**  **(individuals)** | **Oral sucker diameter (OSD) (μm)**  **(min-max)** | **Caudal sucker diameter (CSD) (μm)**  **(min-max)** | **CSD/ OSD** | **Yolk score** | **Departure count (individual)**  **(min-max)** |
| --- | --- | --- | --- | --- | --- | --- | --- | --- | --- |
| Day 0 (eggs) | 455.75±36.53  (363.28-564.97) | 409.33±39.12  (298.46-478.14) | 1.11 | - | - | - |  | - | - |
| Day 1  (hatching) | 563.30±70.76  (411.94-678.34) | 375.53±41.73  (305.87-473.61) | 1.50 | 166,205.72 (5,050.37) | 175.78±24.64  (145.54-224.47) | 246.87±23.41  (197.34-278.74) | 1.40 | 10.00 | 0 |
| Day 2 | 580.81±66.46  (483.47-821.41) | 383.13±28.69  (330.54-444.01) | 1.52 | 174,840.36 (4,800.95) | 181.15±24.69  (145.54-246.67) | 256.14±16.86  (219.54-16.86) | 1.41 | 10.00 | 0 |
| Day 3 | 614.02±66.43  (454.05-770.27) | 422.86±30.01  (348.72-471.79) | 1.45 | 204,007.65 (4,114.55) | 218.28±36.65  (148.00-294.59) | 290.55±21.76  (256.54-351.28) | 1.33 | 10.00 | 0 |
| Day 4 | 683.59±74.99  (540.54-917.14) | 459.47±74.99  (391.89-548.57) | 1.49 | 246,786.09 (3,401.33) | 217.54±35.11  (153.85-271.79) | 342.31±13.85  (305.13-371.79) | 1.57 | 9.94 | 0 |
| Day 5 | 784.81±76.39  (638.89-1,025.42) | 505.14±43.43  (443.06-586.67) | 1.55 | 311,490.91 (2,694.78) | 247.82±39.99  (175.26-309.63) | 404.01±16.34  (360.13-438.82) | 1.63 | 9.94 | 0 |
| Day 6 | 834.61±159.99  (560.00-1,273.33) | 503.76±71.64  (382.86-703.33) | 1.66 | 330,349.24 (2,540.95) | 278.68±35.56  (153.85-345.95) | 366.25±32.67  (287.18-427.03) | 1.31 | 9.74 | 0 |
| Day 7 | 1,059.60±130.22  (853.33-1,503.33) | 576.47±77.43  (463.33-806.67) | 1.84 | 479,933.21 (1,748.99) | 314.59±29.98  (232.43-362.16) | 408.86±57.27  (305.41-551.35) | 1.30 | 8.94 | 0 |
| Day 8 | 1,327.21±143.39  (1,056.00-1,804.00) | 720.62±108.96  (568.00-1,052.17) | 1.84 | 751,471.16 (1,117.01) | 409.85±52.60  (286.67-496.30) | 526.16±81.13  (376.67-722.22) | 1.28 | 8.24 | 0 |
| Day 9 | 1,672.45±330.87  (1,148.83-2,402.09) | 886.78±105.48  (673.08-1,165.87) | 1.89 | 1,165,290.58 (720.34) | 480.08±86.40  (300.75-639.10) | 613.40±118.08  (430.00-920.00) | 1.28 | 7.52 | 0 |
| Day 10 | 2,703.48±746.09  (1,860.47-5,977.44) | 1,457.04±242.73  (1,052.63-2,361.11) | 1.86 | 3,094,991.49 (271.21) | 559.69±116.33  (336.54-740.74) | 683.57±123.63  (516.83-1,045.67) | 1.22 | 6.22 | 0 |
| Day 11 | 3,480.97±564.42  (2,573.73-4,932.98) | 1,778.29±235.38  (1,293.30-2,355.66) | 1.96 | 4,863,704.19 (172.58) | 737.95±158.15  (481.93-963.86) | 936.71±162.51  (696.20-1,376.58) | 1.27 | 5.20 | 0 |
| Day 12 | 3,651.95±487.64  (2,646.10-5,016.23) | 1,454.00±209.24  (1,040.00-2,000.00) | 2.51 | 4,172,089.80 (201.19) | 872.57±181.71  (514.29-1,371.43) | 1,183.46±183.71  (808.27-1,766.92) | 1.36 | 4.40 | 0 |
| Day 13 | 4,274.81±625.46  (3,251.88-5,808.27) | 1,789.95±278.36  (1,244.02-2,392.34) | 2.39 | 6,012,057.07 (139.62) | 932.57±228.77  (571.43-1,628.57) | 1,344.84±259.88  (757.89-1,978.95) | 1.44 | 3.22 | 0 |
| Day 14 | 5,510.72±749.83  (4,164.59-8,129.68) | 2,133.33±219.91  (1,542.29-2,935.32) | 2.58 | 9,237,021.73 (90.87) | 964.41±139.52  (711.74-1,281.14) | 1,558.01±224.43  (1,103.20-2,206.41) | 1.62 | 2.08 | 0.10±0.32  (0-1) |
| Day 15 | 6,608.80±759.02  (5,190.62-7,829.91) | 2,391.01±377.23  (1,741.57-3,146.07) | 2.76 | 12,415,628.85 (67.61) | 1,008.86±153.32  (738.01-1,328.41) | 1,665.90±305.19  (1,187.74-2,375.48) | 1.65 | 1.32 | 1.20 ±1.03  (0-3) |
| Day 16 | 7,600.12±872.87  (5,969.21-9,004.40) | 2,379.77±203.26  (1,622.09-2,459.30) | 3.19 | 14,210,830.63 (59.07) | 953.72±144.94  (697.67-1,255.81) | 2,548.97±254.04  (1,603.45-2,741.38) | 2.67 | 0.60 | 2.20±0.63  (1-3) |
| Day 17 | 8,074.03±1,365.47  (5,888.11-12,086.12) | 2,623.55±520.09  (1,693.70-3,839.06) | 3.08 | 16,643,481.69 (50.43) | 956.21±270.18  (480.16-1,975.52) | 1,712.25±291.22  (1,137.83-2,675.78) | 1.79 | 0.66 | 5.00 ±0.82  (4-6) |
| Day 18 | 8,392.09±1,419.26  (6,120.06-12,562.23) | 2,721.52±539.51  (1,756.95-3,982.43) | 3.08 | 17,945,143.44 (46.78) | 1,020.50±238.34  (512.45-2,108.35) | 2,004.98±341.02  (1,332.36-3,133.24) | 1.96 | 0.42 | 5.80±1.32  (4-8) |
| Day 19 | 9,806.43±1,630.34  (6,497.46-14,517.77) | 3,004.74±660.10  (1,675.13-4,602.37) | 3.26 | 23,151,660.39 (36.26) | 1,165.82±349.57  (558.38-2,436.55) | 2,164.13±438.78  (1,404.40-3,620.98) | 1.86 | 0.14 | 13.80±0.79  (13-15) |
| Day 20 | 10,741.71±1,623.25  (6,994.54-15,628.42) | 3,281.24±676.93  (1,803.28-4,954.46) | 3.27 | 27,693,380.69 (30.31) | 1,269.58±371.50  (1,511.84-3,898.00) | 2,363.21±455.44  (1,511.84-3,898.00) | 1.86 | 0.04 | 21.30±2.79  (19-29) |
| Day 21 | 11,676.24±1,764.47  (7,603.06-16,988.09) | 3,351.13±691.35  (1,841.69-5,059.99) | 3.48 | 30,743,892.43 (30.31) | 1,269.58±371.50  (1,511.84-3,898.00) | 2,363.21±455.44  (1,511.84-3,898.00) | 1.91 | 0.00 | 135.1±35.43  (91-190) |
| Parent | 53,802.04±5,632.71  (49,232.57 -61,045.56) | 26,475.53 ± 4,609.98  (21,218.91- 32,175.33) | 2.03 | Full area: 1,119,200,760.57  ¾ area: 839,400,570.42 | 9,147.65±2,715.93  (6,169.15-12,542.45) | 10,874.05±2,222.81  (8,905.47-13,418.16) | 1.19 | - | - |
