# Supplementary material for: Juvenile detachment, an early sign of departure from parental care, in the leech Orientobdelloides siamensis (Oka, 1917)
Source: PLoS One. 2024 Nov 25;19(11):e0302921. doi: 10.1371/journal.pone.0302921 (PMC11588279; doi:10.1371/journal.pone.0302921)
Supplement: S2 Table — (DOCX) [file pone.0302921.s002.docx]

**Supplementary Table 2** Characteristics and cross-sectional area of each annuli (1-76) from *Orientobdelloides siamensis*

| **Annuli** | **Anterior height (μm)** | **Width  (μm)** | **Posterior height (μm)** | **Cross-sectional area (CSA) (μm)** |
| --- | --- | --- | --- | --- |
| 1 | 29.70±3.86 | 143.14±17.13 | 32.86±3.93 | 205.70±24.86 |
| 2 | 31.91±4.14 | 229.88±27.51 | 35.30±4.22 | 297.10±35.81 |
| 3 | 28.70±3.73 | 197.35±23.62 | 31.75±3.80 | 257.80±31.08 |
| 4 | 44.96±5.84 | 223.38±26.74 | 49.73±5.95 | 318.06±38.43 |
| 5 | 40.34±5.24 | 130.12±15.57 | 44.62±5.34 | 215.09±26.07 |
| 6 | 46.76±6.07 | 130.12±15.57 | 51.73±6.19 | 228.61±27.75 |
| 7 | 51.98±6.75 | 114.94±13.76 | 57.50±6.88 | 224.42±27.29 |
| 8 | 40.14±5.21 | 117.11±14.02 | 44.40±5.31 | 201.65±24.46 |
| 9 | 42.35±5.50 | 91.09±10.90 | 46.84±5.61 | 180.28±21.93 |
| 10 | 48.77±6.33 | 88.92±10.64 | 53.95±6.46 | 191.64±23.34 |
| 11 | 50.38±6.54 | 114.94±13.76 | 55.72±6.67 | 221.04±26.87 |
| 12 | 64.63±8.39 | 88.92±10.64 | 71.48±8.56 | 225.03±27.48 |
| 13 | 51.78±6.72 | 132.29±15.83 | 57.28±6.86 | 241.35±29.31 |
| 14 | 69.24±8.99 | 140.97±16.87 | 76.59±9.17 | 286.80±34.90 |
| 15 | 70.45±9.15 | 117.11±14.02 | 77.92±9.33 | 265.48±32.36 |
| 16 | 76.67±9.95 | 130.12±15.57 | 84.80±10.15 | 291.60±35.54 |
| 17 | 74.46±9.67 | 88.92±10.64 | 82.36±9.86 | 245.74±30.04 |
| 18 | 70.85±9.20 | 104.10±12.46 | 78.37±9.38 | 253.31±30.91 |
| 19 | 79.28±10.29 | 104.10±12.46 | 87.69±10.50 | 271.07±33.11 |
| 20 | 129.46±16.81 | 127.95±15.31 | 143.19±17.14 | 400.60±49.05 |
| 21 | 126.85±16.47 | 140.97±16.87 | 140.30±16.79 | 408.12±49.92 |
| 22 | 130.46±16.94 | 151.81±18.17 | 144.30±17.27 | 426.57±52.16 |
| 23 | 99.35±12.90 | 140.97±16.87 | 109.89±13.15 | 350.21±42.75 |
| 24 | 71.25±9.25 | 114.94±13.76 | 78.81±9.43 | 265.00±32.31 |
| 25 | 84.30±10.94 | 91.09±10.90 | 93.24±11.16 | 268.62±32.87 |
| 26 | 85.50±11.10 | 153.98±18.43 | 94.57±11.32 | 334.05±40.69 |
| 27 | 86.10±11.18 | 177.84±21.28 | 95.24±11.40 | 359.18±43.70 |
| 28 | 84.70±11.00 | 203.86±24.40 | 93.68±11.21 | 382.24±46.45 |
| 29 | 76.67±9.95 | 206.03±24.66 | 84.80±10.15 | 367.50±44.61 |
| 30 | 114.80±14.91 | 190.85±22.84 | 126.98±15.20 | 432.64±52.75 |
| 31 | 110.19±14.31 | 153.98±18.43 | 121.88±14.59 | 386.04±41.13 |
| 32 | 89.51±11.62 | 140.97±16.87 | 99.01±11.85 | 329.49±40.19 |
| 33 | 96.34±12.51 | 164.82±19.73 | 106.56±12.75 | 367.72±44.82 |
| 34 | 96.74±12.56 | 242.90±29.07 | 107.00±12.81 | 446.64±54.25 |
| 35 | 104.37±13.55 | 268.92±32.19 | 115.44±13.82 | 488.73±59.35 |
| 36 | 103.97±13.50 | 292.78±35.04 | 115.00±13.76 | 511.74±62.10 |
| 37 | 83.90±10.89 | 307.96±36.86 | 92.80±11.11 | 484.65±58.69 |
| 38 | 70.65±9.17 | 318.80±38.16 | 78.14±9.35 | 467.59±56.53 |
| 39 | 94.73±12.30 | 333.98±39.97 | 104.78±12.54 | 533.50±64.62 |
| 40 | 105.97±13.76 | 383.86±45.94 | 117.22±14.03 | 607.05±73.52 |
| 41 | 102.36±13.29 | 268.92±32.19 | 113.22±13.55 | 484.50±58.83 |
| 42 | 95.13±12.35 | 206.03±24.66 | 105.23±12.59 | 406.39±49.43 |
| 43 | 128.45±16.68 | 153.98±18.43 | 142.08±17.01 | 424.51±51.89 |
| 44 | 84.10±10.92 | 127.95±15.31 | 93.02±11.13 | 305.07±37.22 |
| 45 | 85.50±11.10 | 229.88±27.51 | 94.57±11.32 | 409.96±49.77 |
| 46 | 73.06±9.49 | 357.84±42.83 | 80.81±9.67 | 511.70±61.83 |
| 47 | 87.91±11.41 | 242.90±29.07 | 97.24±11.64 | 428.04±51.95 |
| 48 | 106.78±13.86 | 190.85±22.84 | 118.10±14.14 | 415.73±50.65 |
| 49 | 89.72±11.65 | 318.80±38.16 | 99.23±11.88 | 507.75±61.50 |
| 50 | 130.46±16.94 | 294.95±35.30 | 144.30±17.27 | 569.71±69.26 |
| 51 | 126.85±16.47 | 344.83±41.27 | 140.30±16.79 | 611.98±74.29 |
| 52 | 93.93±12.20 | 279.77±33.48 | 103.90±12.43 | 477.59±57.93 |
| 53 | 51.38±6.67 | 273.26±32.71 | 56.83±6.80 | 381.47±46.07 |
| 54 | 33.64±4.37 | 362.18±43.35 | 37.21±4.45 | 433.02±52.09 |
| 55 | 34.20±4.44 | 347.00±41.53 | 37.83±4.53 | 419.03±50.42 |
| 56 | 29.22±3.79 | 318.80±38.16 | 32.32±3.87 | 380.35±45.75 |
| 57 | 35.16±4.57 | 320.97±38.42 | 38.89±4.66 | 395.03±47.56 |
| 58 | 42.71±5.55 | 357.84±42.83 | 47.24±5.65 | 447.79±53.93 |
| 59 | 35.89±4.66 | 271.09±32.45 | 39.69±4.75 | 346.67±41.78 |
| 60 | 52.18±6.78 | 320.97±38.42 | 57.72±6.91 | 430.87±51.99 |
| 61 | 50.74±6.59 | 323.14±38.68 | 56.12±6.72 | 430.00±51.87 |
| 62 | 93.93±12.20 | 258.08±30.89 | 103.90±12.43 | 455.90±55.34 |
| 63 | 99.00±12.85 | 290.61±34.78 | 109.51±13.11 | 499.12±60.55 |
| 64 | 88.53±11.49 | 234.22±28.03 | 97.92±11.72 | 420.67±51.08 |
| 65 | 94.07±12.21 | 210.37±25.18 | 104.05±12.45 | 408.49±49.67 |
| 66 | 88.12±11.44 | 229.88±27.51 | 97.46±11.67 | 415.46±50.45 |
| 67 | 88.73±11.52 | 173.50±20.77 | 98.15±11.75 | 360.38±43.87 |
| 68 | 103.73±13.47 | 208.20±24.92 | 114.73±13.73 | 426.65±51.93 |
| 69 | 98.59±12.80 | 199.52±23.88 | 109.05±13.05 | 407.16±49.55 |
| 70 | 95.30±12.37 | 177.84±21.28 | 105.42±12.62 | 378.56±46.10 |
| 71 | 71.07±9.23 | 273.26±32.71 | 78.61±9.41 | 422.93±51.20 |
| 72 | 66.14±8.59 | 251.57±30.11 | 73.15±8.76 | 390.87±47.32 |
| 73 | 54.43±7.07 | 290.61±34.78 | 60.21±7.21 | 405.24±48.94 |
| 74 | 50.94±6.61 | 314.47±37.64 | 56.34±6.74 | 421.75±50.88 |
| 75 | 38.20±4.96 | 299.28±35.82 | 42.26±5.06 | 379.75±45.75 |
| 76 | 47.65±6.19 | 180.00±21.54 | 52.71±6.31 | 280.36±33.94 |
| Average of anterior part (annuli 1-29) | 68.55±28.25  (28.70-130.46) | 137.83±40.98  (88.92-229.88) | 75.82±31.25  (31.75-144.30) | 282.20±69.90  (180.28-426.57) |
| Average of posterior part (annuli 30-76) | 81.25±27.63  (29.22-130.46) | 261.77±67.08  (127.95-383.86) | 89.87±30.56  (32.32-144.30) | 432.90±68.31  (280.36-611.98) |
| Total average (annuli 1-76) | 84.51±31.38  (31.75-144.30) | 241.48±84.03  (88.92-383.86) | 76.41±28.37  (28.70-130.46) | 375.40±100.28  (180.28-611.98) |
